# Mexico’s “*ley de narcomenudeo*” drug policy reform and the international drug control regime

**DOI:** 10.1186/1477-7517-11-31

**Published:** 2014-11-14

**Authors:** Tim K Mackey, Daniel Werb, Leo Beletsky, Gudelia Rangel, Jaime Arredondo, Steffanie A Strathdee

**Affiliations:** Department of Anesthesiology, School of Medicine, University of California, San Diego, CA USA; Division of Global Public Health, School of Medicine, University of California, San Diego, CA USA; Global Health Policy Institute, 8950 Villa La Jolla Drive, San Diego, CA USA; School of Law, Northeastern University, Boston, MA USA; Bouvé College of Health Sciences, Northeastern University, Boston, MA USA; Department of Population Studies, El Colegio de la Frontera Norte, Tijuana, Mexico

**Keywords:** International drug policy, Single convention, Harm reduction, Mexico drug policy, Drug policy reform, UN office of drugs and crime, Global health

## Abstract

It has been over half a century since the landmark Single Convention on Narcotic Drugs was adopted, for the first time unifying international drug policy under a single treaty aimed at limiting use, manufacture, trade, possession, and trafficking of opiates, cannabis, and other narcotics. Since then, other international drug policy measures have been adopted, largely emphasizing enforcement-based approaches to reducing drug supply and use. Recently, in response to concerns that the historic focus on criminalization and enforcement has had limited effectiveness, international drug policies have begun to undergo a paradigm shift as countries seek to enact their own reforms to partially depenalize or deregulate personal drug use and possession. This includes Mexico, which in 2009 enacted national drug policy reform partially decriminalizing possession of small quantities of narcotics for personal consumption while also requiring drug treatment for repeat offenders. As countries move forward with their own reform models, critical assessment of their legal compatibility and effectiveness is necessary. In this commentary we conduct a critical assessment of the compatibility of Mexico’s reform policy to the international drug policy regime and describe its role in the current evolving drug policy environment. We argue that Mexico’s reform is consistent with flexibilities allowed under international drug treaty instruments and related commentaries. We also advocate that drug policy reforms and future governance efforts should be based on empirical evidence, emphasize harm reduction practices, and integrate evidence-based evaluation and implementation of drug reform measures.

## Commentary

### Background

It has been over half a century since the landmark, as amended by the 1972 Protocol amending the Single Convention on Narcotic Drugs 1961, was adopted (Single Convention), for the first time unifying international drug policy under a single treaty designed to limit the use, manufacture, trade, possession, and trafficking of opiates, cannabis, and other narcotic and similar drugs [1]. Since then, other international drug policy instruments have also been adopted, including the 1971 Convention on Psychotropic Substances (CPS) and the 1988 Convention against Illicit Traffic in Narcotic and Psychotropic Substances (Illicit Traffic Convention), aimed at limiting use of narcotic and psychotropic drugs exclusively for medical and scientific purposes while also criminalizing their unauthorized production and trade [1, 2].

More than 50 years later, the success of these widely adopted instruments is in question. An epidemic of global drug use continues, with an estimated 149–271 million illicit drug users reported in 2009 and an overall increase in incarceration for drug-related offenses [3–5]. Critically, drug dependence and injection drug use and its related harms are associated with overdose mortality, drug dependence, and the transmission of HIV and hepatitis B and C viruses [5–7]. As such, the effectiveness of enforcement-based approaches to reducing drug supply and limiting drug use which have been emphasized by a range of United Nations drug control bodies or organs—including the United Nations Office of Drugs and Crime (UNODC), The Commission on Narcotic Drugs (CND), and the International Narcotics Board (INCB)—has been questioned, while concerns exist that a criminalization-based approach exacerbates negative health consequences for people who use drugs [1, 2, 8, 9].

At the same time, support for the international drug control governance regime and the policy approaches codified by these entities and instruments appears to be waning, with at least 30 countries enacting their own alternative approaches through domestic drug policy reform [10]. These include policies of complete and partial deregulation/decriminalization, or decisions by national governments to withdraw from or simply cease to enforce treaty obligations prohibiting personal use and possession of drugs [8, 11]. Despite these attempts at policy experimentation, there is currently lack of sufficient empirical evidence regarding the effectiveness of these reform measures or the impact of their intended and unintended consequences. Reform developments also coincide with ongoing concerns regarding the impact of the international drug conventions on limiting access to pain management medication, use and regulation of cannabis, and a growing evidence base suggesting that less punitive approaches like harm reduction modalities are effective in mitigating negative health consequences of drug abuse [2, 8, 11–17].

One country now at the center of this international debate is Mexico, which in 2009 adopted controversial drug policy reform. Mexico has borne a disproportionate burden of drug-related harms, including an epidemic of drug cartel-initiated violence, a burgeoning prison population, and mounting syndemics related to substance use and infectious diseases [18–20]. Mexico’s law decriminalizes possession of small quantities of narcotics, requires drug treatment for repeat offenders, and shifts responsibility of implementation and legal prosecution from the federal to state level [21, 22]. In this piece, we critically examine Mexico’s drug reform and its compatibility to the international drug policy governance regime, which we adopt to define as the system of formal institutions, legal instruments, norms, and processes that govern global drug control [23]. We argue that the evaluation and generation of empirical data to appropriately assess the impact of this reform is critical, given the evidence that drug-related harms remain pervasive in Mexico and given the potential that this reform may serve to inform future developments in drug policy reform in other settings [18, 19].

### The international drug policy regime

#### Flexibilities and ambiguities

With 184 UN member states having signed on to the Single Convention, this treaty has near unanimous adoption internationally. However, it is not self-executing, meaning that it requires member states to translate and implement treaty-bound obligations through their own national legislation [1, 11]. Additionally, the Single Convention also includes flexibilities due to the confusion created by a lack of definitive treaty terms [1, 11]. This includes ambiguity regarding the specific definition of “medical and scientific” purposes [1, 11].

In response, there have been several attempts to clarify whether the Single Convention creates an affirmative obligation for states to penalize the possession of drugs for personal consumption. Specifically, *Article 36* generally requires states to penalize drug-related offenses, yet it is unclear if the term “possession” contained in this clause refers solely to trafficking of drugs or also encompasses personal consumption/possession, opening up the possibility for policy variation [1, 24]. The *Commentary to the Single Convention* notes that *Article 36* may be interpreted differently by national governments, and if they choose to impose penalties, such penalties need not constitute a “serious offense” and can instead take the form of administrative penalties or fines [1]. In 1977, the UNODC provided a more definitive response to this question, explaining that wholesale legalization of drugs for non-scientific and non-medical purposes was prohibited and unacceptable but also clarifying that countries are under no obligation to impose penal sanctions against unauthorized personal consumption and possession [25].

Similar to the Single Convention, the CPS and Illicit Traffic Convention are not self-executing treaties and face similar issues regarding interpretation and flexibilities [11]. CPS *Article 22* provides qualifiers that limit the scope of its obligations to a country’s constitutional limitations and domestic law and aligns with flexibilities in the *1972 Protocol amending the Single Convention* allowing countries to substitute penal offenses against substance abusers with “treatment, education, after-care, rehabilitation and social reintegration”.

*Article 3* of The Illicit Trade Convention takes a more direct stance to addressing personal possession by specifically calling for the criminalization of possession, purchase, or cultivation for personal consumption under domestic law but also contains similar language limiting treaty obligations to a country’s constitutional principles or “basic concepts of its legal system”, a flexibility that could be broadly interpreted. Further, it remains unclear if *Article 3* is binding on states as it defaults back to obligations of the Single Convention and CPS that do not appear to require penalization.

Overall, commentators such as Bewley-Taylor and Jelsma have noted that decriminalization of possession, purchase, and cultivation for personal use, as well as provisioning of harm reduction services, likely operates within the confines of the international drug control regime [26]. In contrast, policies that create legally regulated markets for non-medical use of scheduled drugs are clearly prohibited by the conventions [26]. Additionally, they note that legal conflict, inconsistencies, and ambiguities continue to exist, supporting further drug policy experimentation [26].

#### Challenges to the international drug control regime

Further complicating treaty interpretations are practical considerations regarding the level of enforceability of the international drug control policy regime. For example, even if a country violates its treaty-bound obligations, drug control bodies erected under the conventions, such as the INCB—the control organ charged with interpretation, monitoring, and implementation of the conventions—have few options with which to confront offending countries [11]. While the treaties allow the INCB Board to reduce a member state’s opium export quotas in response to illicit production or diversion or *recommending* the issuance of export or import bans against violating countries, these are seldom exercised [11].

The Single Convention’s prohibition of cannabis (embedded in Schedules I and IV of the Convention) has also been challenged or ignored by several jurisdictions [27]. Cannabis is perhaps the most high-profile drug subject to legalization, decriminalization, and deregulation efforts at both the national and state levels and has thereby engendered ongoing debate about its reclassification/rescheduling within the treaties via amendment or denunciation of the Single Convention [8, 27]. Specifically, the INCB has formally stated that Uruguay’s national law to legalize and regulate cultivation and sale of cannabis (currently being implemented) is in direct violation of the Single Convention [28, 29]. Similarly, jurisdictions technically not direct parties to the Convention, such as U.S. states of Colorado and Washington, have voted to pass laws to legalize, regulate, and tax cannabis, which the INCB also states is in breach of United States’ commitments under the Single Convention [26, 30].

Countries have also taken more direct action by challenging the international drug treaties through re-accession when faced with incompatible domestic policies that clearly conflict with the Single Convention. In 2012, Bolivia took the unprecedented action of formally withdrawing as a party to the treaty in order to uphold the country’s long-standing tradition of coca leaf chewing, a practice now banned by the Single Convention [11, 31]. Bolivia formally amended its own Constitution to allow its government to challenge the ban, which resulted in a formal request with the CND to amend the convention that ultimately failed due to certain member state objections [11]. Bolivia’s withdrawal was then followed with a request to re-accede to the Convention with reservations (allowed by the treaty) against the ban to allow coca leaf and its traditional uses [11]. Bolivia’s withdrawal and re-accession entered into force on February 2013, when only 15 countries (one-third required) objected to Bolivia’s reservation, then permitted it to be accepted [31].

In contrast to these policy approaches, Mexico’s recent drug policy reform represents an interesting balance between complying with the general legal obligations of these treaties while also developing targeted policy interventions that are compatible with flexibilities discussed.

### Ley de narcomenudeo: Mexico’s drug policy reform

#### Description of the law and controversy

In August 2009, Mexico enacted a drug policy reform known as the Small-Scale Drug Law (“*ley de narcomenudeo*”) amending *Article 478* of the country’s Federal General Health Law [18, 20–22, 32]. The reform follows earlier amendments to the Federal Criminal Code in 1994 that decriminalized certain quantities of narcotics strictly for personal consumption. The new reform also expanded previous legal amendments by eliminating criminal penalties for personal possession of small, specified amounts of cocaine (0.5 g), heroin (50 mg), LSD (0.015 mg), methamphetamine (40 mg), and marijuana (5 g) (see Table 1), as a response to the increased substance use, drug-related violence, and crime that have rapidly escalated in Mexico over the past decade [18, 20, 21, 33]. The law also specifies that following apprehension by police, individuals in possession of sub-threshold amounts will receive a police record stating that they have received “no penal action” and then will be released [18, 20]. However, in the event an individual is apprehended a third time, they will be required to enter mandatory drug treatment, though penalties for non-compliance are not specified.

**Table 1:** **Drugs covered under** ***ley de narcomenudeo***

Drug	Applicable UNODC treaty	Mexico possession limit
Opium	Single convention (Schedule I)	2 g
Heroin	Single convention (Schedule I)	50 mg
Marijuana	Single convention (Schedule I)	5 g
Cocaine	Single convention (Schedule I)	500 mg
LSD	Convention on psychotropic substances (schedule I)	0.015 mg
MDMA	Convention on psychotropic substances (schedule I)	40 mg (powder, granulate, crystal)
		200 mg (one unit tablet or caplet)
MDA	Convention on psychotropic substances (schedule I)	40 mg (powder, granulate, crystal)
		200 mg (one unit tablet or caplet)
Methamphetamine	Convention on psychotropic substances (schedule II)	40 mg (powder, granulate, crystal)
		200 mg (one unit tablet or caplet)

The *ley de narcomenudeo* received both support and criticism for its new approach to domestic drug control including comments from the INCB Board in 2009 that state-based decriminalization policies posed a threat to the coherence of the international drug control regime [34]. Specifically, proponents of the law argued that it would allow law enforcement to focus on the illicit criminal trade and trafficking of drugs, which is a core principle of the Single Convention and other drug control treaties [18, 20, 32]. In addition, the mechanism requiring mandatory drug treatment for repeat offenders has been lauded for requiring the scale-up of much-needed opioid substitution programs [32]. However, opponents have countered that the reform will increase the availability of illicit drugs, encourage use, could lead to more police encounters for drug users, and potentially exacerbate risky drug-related behaviors [18, 22, 35]. In addition, there are concerns regarding Mexico’s capacity to adequately finance and expand access for drug dependence treatment, particularly opioid substitution treatment ostensibly contemplated by the law, and whether the country can ensure the quality and appropriateness of addiction treatment while preventing the mistreatment and abuse of drug users in treatment programs [18, 20].

#### Compatibility with the international drug control regime

Despite its controversy, Mexico’s drug policy reform is innovative in many ways. First, it partially deregulates a select group of illicit drugs that are covered under multiple drug treaty instruments including the Single Convention and the CPS (see Table 1). It deviates from other drug reform measures that have decriminalized the possession of a single drug (e.g., cannabis in Uruguay) or of *all* illicit drugs (e.g., Portugal decriminalizes purchase, possession, and consumption for 10-day personal supply) [13, 36, 37]. Further, though the law does not criminalize individual possession of specified low-threshold amounts on the first or second apprehension, offenders nevertheless receive an administrative record, and a third apprehension triggers mandatory diversion that, in principle, can triage drug-dependent individuals to appropriate treatment [20]. In this sense, the law does not enact an approach of total prohibition nor does it completely decriminalize or legalize personal use and possession, as treatment paired with the possibility of treatment/sanctions is the pathway provided to repeat offenders [8, 38].

While Mexico is a party to all of these international treaties, only lodging limited reservations, the *ley de narcomenudeo* appears to fall within the range of flexibility permitted under the Single Convention, CPS, and Illicit Traffic Convention (see Table 2). Specifically, Mexico’s partial depenalization law with its variation of a “three strikes” system arguably falls under what is permissible under *Article 36* of the amended Single Convention, *Article 22* of CPS, and related treaty commentaries.

**Table 2:** **Mexico’s UNODC treaty adherence status**

Treaty	Number of parties	Treaty entry into force	Mexico’s date of ratification	Mexico’s adherence status
Single convention on narcotic drugs (1961)	184	8 August 1975	27 April 1977	No reservations
Convention on psychotropic substances (1971)	183	16 August 1976	20 February 1975^a^	Indigenous ethic group traditional use of wild plants containing psychotropic substances in Schedule I
UN convention against illicit traffic in narcotic drugs and psychotropic substances (1988)	188	11 November 1990	11 April 1990	Reservation against USA unilateral claim to justification for denying legal assistance to a State that requests it

^a^Accession.

Beyond its compatibility with the existing international drug legal regime, we contend that the success of Mexico’s drug policy reform relies on a number of key developments. These include the successful implementation of a large-scale and effective addiction treatment system; the meaningful involvement and training of law enforcement and the judiciary in triaging individuals to treatment, monitoring, and evaluation; and improved governance to address corruption and ensure ethical enforcement/treatment [39]. This specifically includes assessment of the implementation of the reform law by local jurisdictions (given that the implementation of *ley de narcomenudeo* is left up to the state and local governments), and periodic re-evaluation of the law and its requirements.

Within this context, it may be necessary to reassess some aspects of the drug policy reform to ensure its effectiveness. For example, it may be necessary to revise the law’s personal drug possession thresholds, as they do not appear to be scientifically based and are of low enough quantities that they could increase the likelihood of police extortion or misclassification of traffickers [40]. Further, procedural issues requiring police to take confiscated drugs to authorities for weighing against threshold amounts introduce inefficiencies and also potential occupational health hazards that should be addressed.

In addition, adequate scale-up of accessible and affordable drug treatment, such as the expansion of methadone maintenance treatment (MMT) under the *Consejo Nacional contra las Adicciones* federal drug control program in Mexico, will be necessary to provide capacity for an influx of cases of opioid-dependent individuals [32]. However, continuing barriers in scaling up MMT, low levels of MMT enrollment possibly influenced by discretionary policing practices, and continued nationwide arrests of individuals for drug possession even after passage of the law indicate that these challenges have yet to be adequately addressed [20, 39]. Some areas of the country, such as the state of Baja California with the highest number of mandatory drug treatment referrals, appear to be making progress, although it is slower than anticipated [39].

### Reform: the need for evidence-based policymaking and implementation science

The international drug control regime is undergoing a paradigm shift. Nevertheless, ambiguity and conflict with institutions such as the INCB remain regarding the extent of the flexibilities allowed by the international drug treaties in relation to these state-based measures [1, 11, 24, 34]. Beyond national efforts to reform drug policy, signs of change are also evident at the international level, with calls for the redirection of anti-drug efforts towards evidence-based models of harm reduction reified by the Vienna Declaration and by the Harm Reduction Coalition and Open Society Foundations [8, 41, 42]. A working group of the UNODC also recently announced groundbreaking recommendations explicitly stating that criminal sanctions are not beneficial for addressing drug dependence [43].

As an increasing number of countries, such as Mexico, enact alternatives to enforcement-based approaches to drug use, international drug control organizations should recognize the opportunities of existing treaty flexibilities for policy experimentation and the need to better assess the implementation, effectiveness, compatibility, and impact of these reform measures on a range of health, economic, and societal outcomes. This should include meaningful support, funding, and prioritization of public health, substance abuse, and policy/legal research examining whether reform measures are properly implemented and have their desired effect of reducing substance abuse-related health risks while also enabling law enforcement to focus on the criminal element of the drug trade [2, 7, 8, 41]. Some suggested research priority areas which are outlined in Table 3.

**Table 3:** **Proposed research priority areas for drug reform**

Theme	Description
Implementation science	Development of implementation science approaches, defined as the “study of methods to promote the integration of research findings and evidence into healthcare policy and practice” [44], to provide dissemination and incorporation of research about the successes and challenges of varying drug policy reform practices, including the *ley de narcomenudeo.*
Comparative policy analysis	Detailed exploration of the association between decriminalization policies and reductions in drug use using cross-comparisons of multiple jurisdictions and different reform strategies [45].
Development of indicators	Establish a set of effectiveness indicators that go beyond conventional metrics regarding the size and composition of illicit drug markets and prevalence of use. Instead, such metrics should incorporate a range of outcomes that measure drug-related harms such as the transmission of blood-borne diseases among people who inject drugs, the incidence of non-fatal and fatal overdoses, and emergency room mentions of drugs [9].
International development on evidence-based policy mechanisms	Based on approaches above, cooperation on building international consensus and development of culturally appropriate evidence-based policy mechanisms that are compatible/align with existing treaty flexibilities. Internationally agreed upon best practices guidelines for implementation of these policy measures should also be pursued along with avenues for technical assistance [41].
Multidisciplinary/multi-sector research partnerships	Key for development of formalized partnerships between local, national, and international public health departments, law enforcement officials, and policymakers to work closely with impartial experts to collect and evaluate data to guide future evidence-based approaches to address drug abuse, promote harm reduction, and ensure human rights protection.

Inevitably, some may question whether new drug policy reform models should be pursued by individual countries in the absence of robust empirical data and policy evaluation that could support their effectiveness and address implementation challenges. However, natural-drug policy experiments have been ongoing for some time, as is the case in Portugal, Switzerland, and the Netherlands, with data on the effectiveness and how to improve these policy alternatives still insufficient but nevertheless emerging [37, 40]. Hence, it is key for local, national, and international stakeholders to examine evidence emanating from drug policy reform measures that operate *both* within the confines/limitations of international drug control law and those reform measures that go beyond these flexibilities. This is necessary to determine what elements of reform and implementation are successful and consistent with public health and harm reduction practices as well as identifying those that pose unintended risks [37, 40].

As drug policy reforms proliferate, this movement will put increasing pressure on the international drug policy community to diligently evaluate emerging models and their impact on indicators of drug-related harm. This can be accomplished by recognizing the urgent need for research to inform future evidence policy-making efforts at the upcoming UN General Assembly Special Session (UNGASS) on Drugs scheduled for 2016 [46]. The UNGASS on Drugs will provide Member States the opportunity to reassess progress towards the “UNODC Political Declaration and Plan of Action on International Cooperation Towards an Integrated and Balanced Strategy to Counter the World Drug Problem” and has been highlighted by Latin American leaders as a forum for debate on alternative approaches to the international drug control regime [46].

### Conclusion

The success or failure of Mexico’s drug policy reform will have broader implications for the evolution of global drug policy reform and its relationship with the international drug control system. Others have called for revisiting, amending, developing new instruments, or withdrawing from international drug policy treaties [1, 8]. In this commentary, however, we argue that existing flexibilities allow for important policy experimentation that needs to be critically researched and evaluated to inform effectiveness and proper implementation. As the momentum for innovative drug policy reform grows, the international drug control regime should modernize by building evidence-based approaches to guide future efforts while also promoting harm reduction and protection of human rights. An international commitment to determine whether these policy experiments are successful is therefore urgently needed in order to provide policymakers across the globe with a set of tools by which to design evidence-based approaches to meaningfully address ongoing drug use, addiction, and its negative health impacts.

## Abbreviations

CND: Commission on Narcotic Drugs
CPS: Convention on Psychotropic Substances
Illicit Traffic Convention: Convention against Illicit Traffic in Narcotic and Psychotropic Substances
INCB: International Narcotics Board
Single Convention: Single Convention on Narcotic Drugs
UNGASS: UN General Assembly Special Session on Drugs
UNODC: United Nations Office of Drugs and Crime
WHO: World Health Organization.
